# Hæmatocele

**Published:** 1851-03

**Authors:** R. W. Hall

**Affiliations:** Hawk Eye, Iowa


### ARTICLE IV.

*Hæmatocele*, By Dr. R. W. Hall, of Hawk Eye, Iowa.

At about 11 o'clock P. M., September last, I was called to go in haste 1 1-2 miles, to see Mr. E. M., who was represented to be in a dangerous condition, as he was suffering most intensely. Upon my arrival, I found him looking rather pale, cool, quiet, and cheerful ; but with agony depicted in his countenance. I inquired for the cause of his suffering, when he answered : " Oh, sir, I am in a bad fix, I have had a general cold, and as I got better better of it, it settled here ; (placing his hand on the pubis,) and as my cold mended, this got worse. I have suffered very much—I have a great deal of pain."

On examination I found the right hemisphere of the scrotum hot, swollen, and somewhat tender ; the integuments stretched, but not very tightly, the tunica vaginalis appeared very tense and hard, the spermatic cord, of the right side, also swollen and hard. The right side of the scrotum overlapped the left, and would measure about 20 inches in circumference, while the left side was but little enlarged. About four days previous to my nightly visit, (but after the swelling had made considerable progress,) he had fatigued himself by running, while driving cattle. After this, the swelling and pain increased much more rapidly. Some 30 or 35 hours before I saw him, a point, in the anterior portion of the tumor, had yielded to the force of the accumulation within, from which some blood, both clotted and fluid, had escaped. This gave some relief, but the aperture becoming partially closed by a clot, and the blood being no longer permitted to escape, (except a very slight oozing of serum,) the swelling and pain returned, and continued to augment, up to the time of my seeing him.

I proceeded at once to enlarge the opening, to the extent of an inch and a half, probably more, but the clot refused to be discharged. I now made a close examination of the mass, both in its position, and of the small parts of it that I was able to dislodge, and could distinctly see fibrinous shreds and filaments passing in all directions, but mostly from the tunica vaginalis toward the centre, in the form of small pillars, with their bases resting on this tunic, and to which, also, the whole mass seemed to adhere, thus showing a tendency to organization. The testicle was pushed as far posteriorily as it could get.

With a suitable probe, I broke up the mass and adhesions, as well as I could, to the distance of two inches or more, by passing it through in every direction ; after which, some small portions were removed. This by removing the tension, gave some ease. I then applied cold wet cloths, and left him.

At a pretty early hour next morning, I visited him again and found the tumor but little diminished in size, except near the orifice : while from it, there still issued some blood. It was, however, more cool and soft, and less painful ; and, the swelling of the spermatic cord was much less prominent. After removing that part of the mass which led toward the source of the broken vessel, viz : toward the symphysis pubis, I injected by means of a small syringe, a solution of sulph. zinc, in that direction. The application of this astringent, once or twice repeated, aided by the cold cloths, soon caused the flow of blood to cease. Most of the clotted mass was now removed. The clot, which was easily removed by pieces, (together with sanious fluid,) appeared to be made up of two distinct portions—one as a jelly of mashed fibrin and blood and the other, which I suppose to be parts of the original mass, not broken up by the probe, was rather more compact, firm, and tenacious, than clotted blood usually is. The cold cloths were continued.

I saw him the following day : there had been no more bleeding, the pain was gone, the swelling had nearly disappeared, and in two or three days more, the patient was on foot, at his usual avocation.

It may be asked, why I did not turn out the whole mass at once ? and why I did not use the injection on my first visit ? To answer the last question first, I could not had it been my wish, for I had not the means, (i. e. no syringe at hand), and as the bleeding was slow there was no immediate danger of fatal hæmorrhage, and *perhaps* the cold application *might* arrest it ; and farther, as the bleeding was small, and "heat, pain, redness, and swelling," were all present, in a greater or less degree, it *might* be well to defer the injection until some of these "symptoms," if not the whole of them, could be at least, partially subdued.

To answer the first, knowing his need, as well as his desire to get about quickly, I did not think it best to open the scrotum its whole length, and thus inflict a larger wound than was actually necessary ; for other things equal, the longer the cut, the more time to heal it ; and, as the contents of the tumor from their nature, could not be urged through a small opening, I removed at first only as much as I could through such an opening without doing violence to the parts. And had the clot been displaced, without a corresponding contraction of the covering membranes immediately following, a new clot would have been the result, unless the broken vessel had been permanently closed. And had the injection been then used, it would have spread over a greater surface of the vaginalis than would have been desirable, and would not have exercised any greater hæmostatic influence, than a smaller quantity applied only to the required point. The sequel I think, justified the delay ; for, after the clot and its adhesions were broken up, and the wasting blood freshly diffused throughout the mass, which evidently softened it, there was little danger and no difficulty attending its removal.

